# Field-free topological behavior in the magnetic domain wall of ferrimagnetic GdFeCo

**DOI:** 10.1038/s41467-021-25926-4

**Published:** 2021-09-23

**Authors:** Zhuolin Li, Jian Su, Shi-Zeng Lin, Dan Liu, Yang Gao, Shouguo Wang, Hongxiang Wei, Tongyun Zhao, Ying Zhang, Jianwang Cai, Baogen Shen

**Affiliations:** 1 Beijing National Laboratory for Condensed Matter Physics, Institute of Physics, Chinese Academy of Sciences, 100190 Beijing, China; 2 School of Physical Sciences, University of Chinese Academy of Sciences, 100049 Beijing, China; 3 Theoretical Division, Los Alamos National Laboratory, Los Alamos, NM 87545 USA; 4 Department of Physics, Beijing Technology and Business University, 100048 Beijing, China; 5 Institute of Advanced Materials, Beijing Normal University, 100875 Beijing, China; 6 Songshan Lake Materials Laboratory, Dongguan, 523808 Guangdong, China

**Keywords:** Spintronics, Magnetic properties and materials

## Abstract

Exploring and controlling topological textures such as merons and skyrmions has attracted enormous interests from the perspective of fundamental research and spintronic applications. It has been predicted theoretically and proved experimentally that the lattice form of topological meron-skyrmion transformation can be realized with the requirement of external magnetic fields in chiral ferromagnets. However, such topological transition behavior has yet to be verified in other materials. Here, we report real-space observation of magnetic topology transformation between meron pairs and skyrmions in the localized domain wall of ferrimagnetic GdFeCo films without the need of magnetic fields. The topological transformation in the domain wall of ferrimagnet is introduced by temperature-induced spin reorientation transition (SRT) and the underlying mechanism is revealed by micromagnetic simulations. The convenient electric-controlling topology transformation and driving motion along the confined domain wall is further anticipated, which will enable advanced application in magnetic devices.

## Abstract (web summary)

Merons and Skyrmions, two topological spin-textures, have attracted a lot of interests due to their potential use in information storage. Here, the authors demonstrate the transformation of Meron pairs into Skyrmions without an applied magnetic field within domain walls of GdFeCo films.

## Introduction

Topological magnetic textures including (anti)meron^1–3^, (anti)skyrmions^4–10^, biskyrmions^6,11^, and bobbers^12^ in the form of vortex^13,14^ spin configurations represent a promising direction for fundamental research and future spintronics. Their nanometer size and electric-current detection/manipulation/generation behavior endow them as competitive candidates for encoding the information bits with dense integration and energy efficiency in the next generation spintronic devices. By exploring and tuning the dedicated interplay among spin–orbit coupling and the competition of magnetic interactions, the materials hosting these topologically nontrivial magnetic textures have been extended from initial chiral magnets with Dzyaloshinskii–Moriya interaction (DMI) to diversified ferromagnets with different stabilization mechanisms^15–17^. Currently, the transverse motion of skyrmions in response to external electric current drive in ferromagnets, known as skyrmion Hall effect^9,18–20^ is regarded as a hurdle in promoting skyrmion applications in racetrack memories, where it is preferable for skyrmions to move along the racetrack. Theoretically, compensated antiferromagnets with two equivalent but antiparallel magnetic subsystems can perfectly cancel the transverse motion and allow skyrmions to move along the electric current direction^21–23^. However, it is hard to experimentally visualize and study intrinsic antiferromagnetic (AFM) skyrmions by using the standard imaging methods because of their net-zero magnetization. Therefore, it is of great importance to figure out distinctive features and properties of the topological textures in ferrimagnetic materials with partially compensated magnetic moments^24,25^.

Due to the compatibility with standard spintronic devices, magnetic thin films or multilayers present good application possibilities, where magnetic bubbles, skyrmions, vortices, and together with magnetic domain walls can be easily tuned by balancing the ferromagnetic exchange, spin anisotropy, and dipolar energy through layer thickness and deposition conditions^26–28^. Recently the topologically nontrivial meron^29^ and skyrmions^30–33^ localized in magnetic domain walls have attracted considerable interest. The intrinsic canted spin configuration and the confining potential in the domain wall might advance the specialties of these magnetic textures and restrict them to move only within the domain wall. In this way, the detrimental skyrmion Hall effect can be avoided. The amorphous ferrimagnetic GdFeCo films^34–36^ regain the focus in skyrmion generation and manipulation due to the antiferromagnetically coupled sublattices^37^ and possible DMI^35^. In this work, we demonstrate the topological transformation between meron pairs and skyrmions in the domain wall via the temperature-induced spin reorientation transition (SRT) and their electric control capability, which does not require any external magnetic field unlike that in chiral lattices^1,2,38^.

## Results

### The temperature-induced Meron–Skyrmion transformation in the domain wall without external magnetic fields

**Fig. 1:**
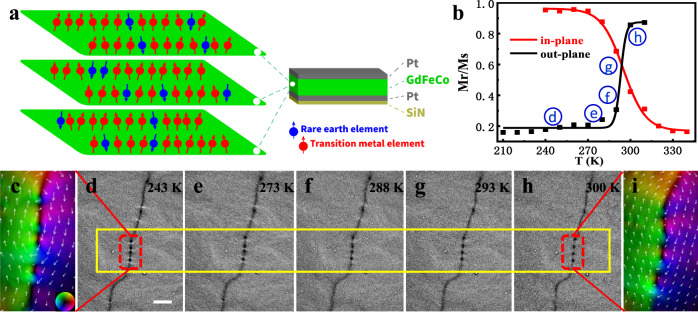
The magnetic domain evolution in amorphous ferrimagnetic GdFeCo film. **a** Schematic illustration of the layer structure. **b** The temperature dependence of the reduced remnant magnetization acquired from *M*–*H* measurements in Gd_15+*x*_(Fe_94_Co_6_)_85−*x*_ (*x* = 0.2), showing the SRT between in-plane and out-of-plane magnetization at about 290 K. **c** The lateral magnetic component distributions deduced from the selected domain wall region at 243 K by using the transport-of-intensity equation (TIE) analysis. The color wheel for the direction of in-plane magnetization is displayed in the inset. **d**–**h** Over-focus L-TEM images at different temperatures showing the corresponding domain wall evolution with increasing temperature. **i** The magnified lateral magnetic component distributions by TIE demonstrating the decreasing of the in-plane magnetization beside the domain wall at 300 K. The scale bar is 2 μm.

The ferrimagnetic amorphous Gd_15+*x*_(Fe_94_Co_6_)_85−*x*_ (*x* = 0.7, 0.4, 0.2, 0) films with thickness about 40 nm are prepared with different RE-to-TM ratios in composition to tune its perpendicular magnetic anisotropy (PMA) (Supplementary Figs. 1 and 2). The SRT temperature increases with *x* in Gd_15+*x*_(Fe_94_Co_6_)_85−*x*_ and the topological transformation can be adjusted to near room temperature for convenient applications (Supplementary Figs. 3 and 4). Figure 1a shows the schematic layer structure with different orientations of TM and RE magnetic moments. The SRT is introduced by the magnetic competition between PMA and shape anisotropy^39^ while changing the temperature. The SRT manifests abrupt spin anisotropy change near 290 K as shown in the reduced remanence magnetization (Fig. 1b), which is extracted from the M-H curve of Gd_15+*x*_(Fe_94_Co_6_)_85−*x*_ (*x* = 0.2). It has been known that the topological nature of the magnetic texture is intimately related to the spin anisotropy with (anti)merons^1–3^ usually in the magnets with easy plane anisotropy and skyrmions/bubbles in magnets with easy axis anisotropy^8,17,18^. The internal magnetic structure evolution inside the domain wall due to the SRT is unraveled by in-situ L-TEM images (Fig. 1d–h). The in-plane magnetization distribution on both sides of the selected domain wall is reconstructed from the transport-of-intensity equation (TIE) analysis (details in the “Methods” section) and is displayed in Fig. 1c and i, respectively. Here, the white arrows aligning antiparallel on the sides of the domain wall demonstrate the local opposite in-plane magnetization component distribution. Since TIE analysis are performed at the same condition, the smaller arrows in Fig. 1i than those in Fig. 1c indicates the in-plane magnetization component tends to point to out-of-plane, which corresponds well with the SRT (Fig. 1b). The significant feature along the domain wall is the disappearance of the white contrast while increasing the temperature.

**Fig. 2:**
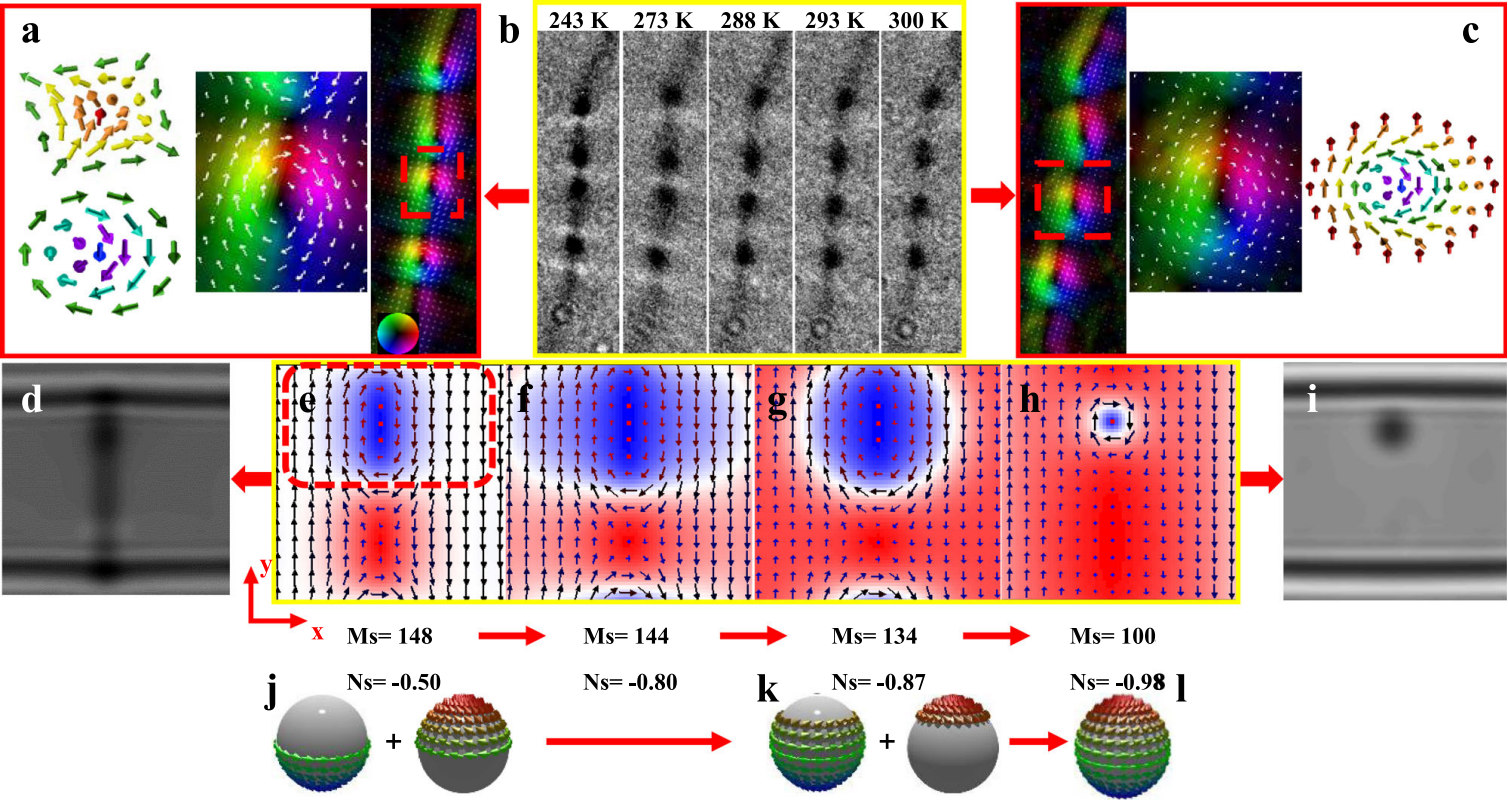
The topology identification of the spin textures in the domain wall of the thin film. **a** Schematic meron pair and the lateral magnetic component distributions were deduced from the selected domain wall region at 243 K by using the transport-of-intensity equation (TIE) analysis. The color wheel for the direction of in-plane magnetization is displayed in the inset. **b** The magnified images from the selected domain wall demonstrating the evolution from meron pairs with alternated dark and white contrast into skyrmions with single dark contrast in the domain wall at 300 K. **c** The in-plane magnetic component distributions and schematic view of skyrmions deduced from the selected domain wall at 300 K. **d** and **i** The corresponding L-TEM contrast simulation. **e**–**h** The simulated domain wall evolution from the meron pair to skyrmion. The value of skyrmion number (*N*_s_) is integrated across half distance of *y*-direction (the dotted rectangle in **e**) and the value ranging from − 0.5 to − 0.98 confirms the topological transformation from meron to skyrmion. The out-of-plane magnetization is represented by regions in red (+*m*_*z*_) and blue (−*m*_*z*_), whereas the in-plane magnetization is represented by white regions with black arrows. **j**–**l** The corresponding schematic stereographic projection of spin textures. The axis in the left corner stands for the corresponding direction used in our main text.

We then focus on the dedicated spin textures in the domain wall in Fig. 2. The magnetic textures with dark/white contrast at the temperature of 243 K correspond to meron pairs (Fig. 2a). With the diminishing of the white contrast in the domain wall, the well-separated dark dot domains at 300 K are identified as skyrmions (Fig. 2c). It should be noted that the domain walls with opposite contrast have the equivalent topological transformation as shown in Supplementary Fig. 3. The size of the topological textures is about 200 nm, which is comparable to the size of the domain wall. Pictorially, a meron can be regarded as a half skyrmion (Schematics in Fig. 2j). A meron has two internal degrees of freedom^3,40^: the polarity, *p*, which is defined as the direction of the spins at the core of the meron, and the standard vorticity, *w*. The topological charge of a meron is given by *N* = *pw*/2, where *p* = +1 for up and *p* = −1 for the down polarity of the cores and *w* = ±1. To determine the topology of the spin texture in Fig. 2a, it is important to identify the spin direction at the core of these magnetic textures, which is unfortunately not accessible by L-TEM. The core polarities of meron pairs have four possibilities which are clarified into two categories (up/up or down/down, up/down, or down/up). If the core polarities of meron pairs are the same (up/up or down/down), their core spin polarization simultaneously tends to either parallel or antiparallel to the out-of-plane spin component in the wall when the out-of-plane easy-axis anisotropy increases across the SRT. That means the pairs will stay or disappear simultaneously when increasing the temperature in contrast to the disappearance of white contrast and the preservation of black contrast in our experiment. Combining with the fact that the final state ends up with magnetic nontrivial textures in our experiment, we propose the opposite core polarity for these spin textures and the same topological charge *N* = −1/2 (meron pair) or *N* = 1/2 (antimeron pair)^3,40,41^. Here, we only demonstrate the spin configuration of the meron pair to present an equal possibility of antimeron pair. When the spin anisotropy is flipped from in-plane to out-of-plane near the SRT, the meron with core polarity antiparallel to the out-of-plane spins remain stable and evolve into skyrmions with topological charge −1, which is well corroborated in the following simulations (Fig. 2e–h). We confirm the reversible transformation between meron pairs and skyrmions in the domain wall while changing the temperature in several heating/cooling cycles. Different from the reported Néel-type skyrmions in sandwiched GdFeCo thin film (5 nm) with interfacial DMI^42^, the skyrmions here are identified as Bloch-type (Fig. 2c) in the relatively thick GdFeCo film (40 nm) (Supplementary Figs. 5 and 6 in Supplementary Note 3). The swirling spin configuration of skyrmions and the meron pairs originate naturally from the helical spin orientation across the domain wall in this centrosymmetric system in correlation with the absence of DMI (Supplementary Figs. 7 and 8 in Supplementary Note 4).

### Simulations for the topological transformation in the domain wall

The numerical simulations are conducted by using the object-oriented micromagnetic framework (OOMMF) software. The in-plane easy axis anisotropy at 243 K can be inferred from the ripple domain and the elongated meron or skyrmion configurations, where the region with spins aligning along the easy axis direction is longer compared to that perpendicular to the easy axis. Also note that domain walls become unstable without the in-plane easy axis anisotropy, because of the in-plane spin rotation symmetry. Across the SRT, an extra out-of-plane easy-axis anisotropy develops and increases with temperature. The above consideration motivates us to introduce the following spin anisotropies ${H}_{{\rm {A}}}=-{A}_{y}{S}_{y}^{2}-{A}_{z}{S}_{z}^{2}$. The spin anisotropy in ${H}_{\rm {{A}}}$ supports four degenerate domains which we denote by (±, ±). The first/second sign represents the *y/z* component of the spin in the domain. Domain walls can be generated between any pair of these four degenerate states. The antiparallel in-plane magnetization component near each side of the domain wall (Fig. 1c) is used for simulation. Since the out-of-plane component of the spin cannot be determined directly in L-TEM, let us first consider the domain wall configuration with parallel out-of-plane components of the spins in these two domains. The corresponding Bloch-type domain wall is verified in experiments and simulations (Supplementary Figs. 5 and 6). The meron pairs are generated from the fine structures with the right and left-hand Bloch wall pieces divided by Bloch lines (Supplementary Figs. 9 and 10). The simulation results also show an opposite core polarity between a meron pair since the domain walls aside from the Bloch lines have different chirality.

These periodic Bloch lines generated in the in-plane Bloch domain walls are different from those in a perpendicular bubble or stripe domains^43–46^. The skyrmion number (*N*_s_) integrated over the *x*-direction and the top half part along *y*-direction of the simulation images in Fig. 2e–h (marked out by the dotted rectangle) is defined as^7^

1 $${N}_{s}=\frac{1}{4\pi }\iint {{{{{\bf{n}}}}}}\cdot \left(\frac{\partial {{{{{\bf{n}}}}}}}{\partial x}\times \frac{\partial {{{{{\bf{n}}}}}}}{\partial y}\right){{\rm {d}}x{\rm {d}}y}\,$$

where **n** is the direction vector of magnetization. The *N*_s_ of a meron is −1/2 despite that the spins away from the meron core are parallel to the domain wall. When the out-of-plane easy axis anisotropy increases across the SRT, the meron with core spin polarization in the same direction (red +*m*_*z*_) as the out-of-plane spin component becomes unstable and shrinks, because there is no energy barrier separating this type of meron and the fully polarized state. At a stronger out-of-plane easy-axis anisotropy, this type of meron disappears completely.

For the core spin polarization in the opposite direction (blue −*m*_*z*_) in the wall, the meron remains stable and evolves into a full skyrmion through continuous growth of the topological charge from −1/2 to −1 (Fig. 2e–h), as illustrated by schematics (Fig. 2j–l). The simulated images with decreasing *M*_s_ and increasing *K*_u_ values (Fig. 2e–h) and the corresponding domain wall contrast simulation (Fig. 2d and i) agree well with the experimental domain wall evolution of Gd_15+*x*_(Fe_94_Co_6_)_85−*x*_ (*x* = 0.2) at different temperatures (Fig. 2b). For other possibilities with both opposite in-plane and out-of-plane magnetization moments on each side of the domain wall (Supplementary Fig. 11), the spin configuration on the edge of a continuous domain wall presents significant differences. Accordingly, the simulated domain wall contrast does not agree with the contrast evolution in L-TEM images (Fig. 1).

**Fig. 3:**
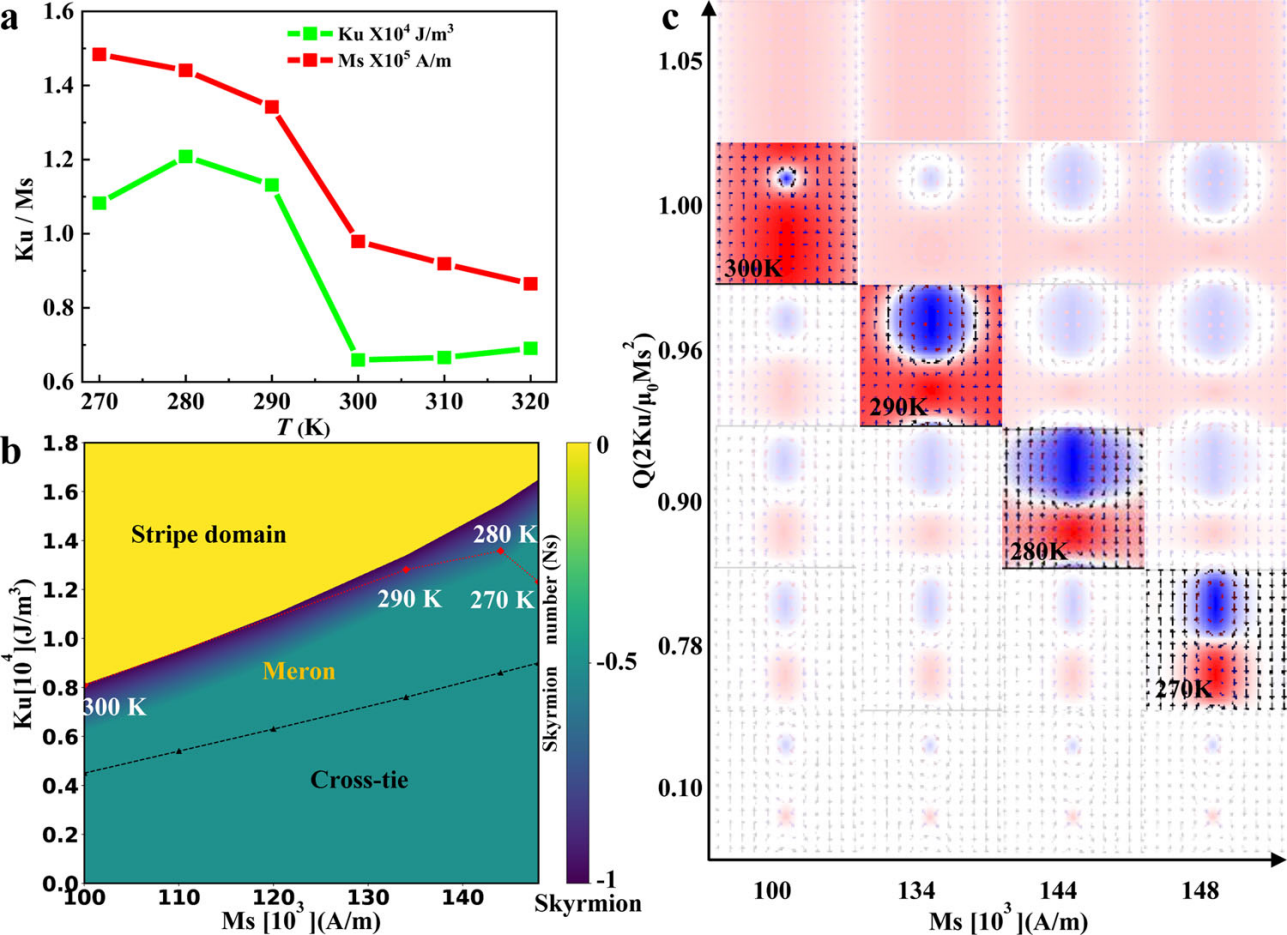
The magnetization and domain evolution based on experiments and simulations in Gd_15+*x*_(Fe_94_Co_6_)_85−*x*_ (*x* = 0.2). **a** The dependence of magnetic parameters on temperature extracted from experimental results. **b** The domain phase diagram in terms of varying *M*_s_ and *K*_u_ for a fixed *A* = 7 × 10^−12^ J/m with experimental data points marked in red. Color map represents the skyrmion number and delineates the boundary between different magnetic phase regions, indicating the continuous transformation between meron pair and skyrmions but the abrupt transition from skyrmion to stripe domains. **c** The simulated magnetization states for different parameters. The highlight images reflect the experimental transformation at different temperatures. The out-of-plane magnetization is represented by regions in red (+*m*_z_) and blue (−*m*_z_), whereas the in-plane magnetization is represented by white regions with black arrows.

The simulation parameters are based on the experimental results of Gd_15+*x*_(Fe_94_Co_6_)_85−*x*_ (*x* = 0.2) (Fig. 3a). The overall domain wall structures in the *K*_u_−*M*_s_ phase diagram are summarized in Fig. 3b with the data points at different temperatures marked out as red rhombic points. Considering the effects of the added uniaxial in-plane anisotropy in our simulation, the out-of-plane anisotropy is slightly larger than in experiments. The transformation between meron and skyrmions is expected near *Q* = *K*_u_/2*πM*_s_^2^ = 1 due to the easily tuned magnetization moments in the canted state, which agrees well with the skyrmion generation condition reported in the previous studies^4–9^. Merons and skyrmions exist in a proper range of *K*_u_ and *M*_s_ with a narrow transition region between them, corresponding to the SRT in our experiments. When *K*_u_ is increased, the skyrmion number gradually changes from meron with *N*_s_ = − 0.5 to skyrmion with *N*_*s*_ = −1 and then abruptly jump to stripe domain state with *N*_*s*_ = 0. Figure 3c shows the detailed simulated magnetization states for different magnetic parameters. To focus on the evolution of the meron pair located in the domain wall, we only present the central part of these simulation images and leave the complete parts listed in Supplementary Note 5. The highlighted images correspond to the experimental transformation at different temperatures. The Néel-type cross tie domain wall remains in the bottom of the phase diagram (Fig. 3b) where the in-plane anisotropy is dominant. It should be noted that no DMI and external magnetic fields are added in the simulations. The intrinsic noncollinear spin textures in the confined domain wall and the spin anisotropy change due to SRT are responsible for the topology transformation between meron pairs and skyrmions, which could be understood better by domain wall topology theory^30,31^.

### Electric control of domain wall topology without magnetic field

**Fig. 4:**
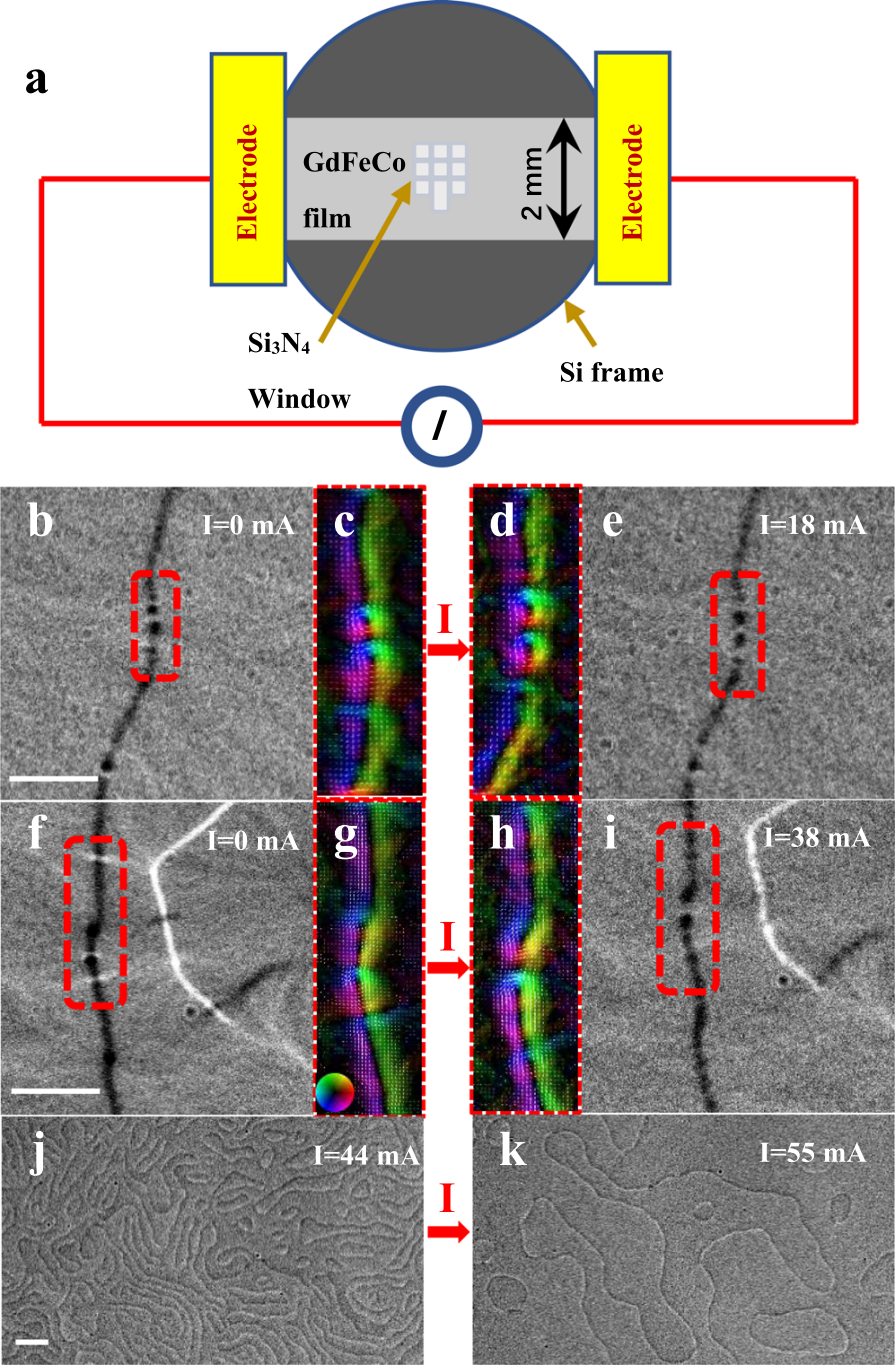
The meron–skyrmion transformation in the domain wall driven by an electric current at room temperature. **a** Schematic illustration for electric current manipulation. **b**–**e** The disappearance of white contrast of L-TEM images and TIE analysis showing the domain wall transformation from merons to skyrmions under the electric current of 18 mA in Gd_15+*x*_(Fe_94_Co_6_)_85−*x*_ (*x* = 0.4). **f**–**k** The magnetic domain evolution under the electric current of 0, 38, 44, and 55 mA in Gd_15+x_(Fe_94_Co_6_)_85−*x*_ (*x* = 0.7), respectively. The enlarged domain wall textures with in-plane magnetization for selected domain wall regions in **c**, **d**, **g**, **h** manifest the possible topology transformation. The color wheel for the direction of in-plane magnetization is displayed in the inset. The scale bar is 1 μm.

**Fig. 5:**
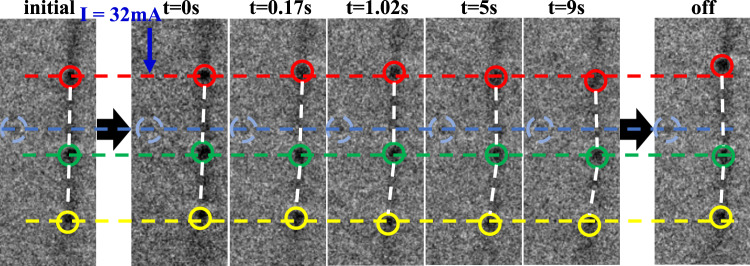
The skyrmion motion in the domain wall driven by an electric current at room temperature in Gd_15+*x*_(Fe_94_Co_6_)_85−*x*_ (*x* = 0.7). L-TEM images showing the skyrmion motion after turning on the electric current of 32 mA for the different time periods, respectively. The blue circle for the fixed defect is marked out to identify the relative movement of the skyrmions on the domain wall. The relative movement of the three skyrmions is demonstrated with different colors, respectively.

The significant transverse deflection of electric-driven skyrmions even within a patterned narrow path^19^ is known as the skyrmion Hall effect in a ferromagnet. The ferrimagnetic materials might offer platforms to minimize the skyrmion Hall effect due to the partially compensated magnetization^35,42,46^. Because of the confining effects for domain wall skyrmions, the only movement along the wall is allowed, thereby, avoiding the detrimental skyrmion Hall effect. Theoretically, the skyrmion motion guided by the domain wall is verified in the simulation (Supplementary Video 1). In experiments, pinning of skyrmions due to the defects in materials can be significant, which requires a more systematic study in preferable nano-fabricated films. Here, the preliminary experiments with electric control behavior in the uniform large sample have promisingly shown that the electric current not only can initiate the SRT and the transformation from merons to skyrmions (Fig. 4) but also can induce the skyrmion motion in the domain wall (Fig. 5 and Supplementary Video 2). The less significant skyrmion movements along the domain wall in experiments than simulations may be attributed to the disorder and pinning effects which are not included in our simulation. It has been verified that electric manipulation can increase the perpendicular anisotropy^47^, switch the magnetization, drive skyrmion movements via spin-transfer^5^ or spin–orbit torque (SOT)^6^. Here, we leave the underlying mechanism of the current induced SRT and skyrmion motion in nano-fabricated films for future study.

## Discussion

We have observed the direct topological transformation between meron pair and skyrmion in the domain wall of amorphous ferrimagnetic GdFeCo, which is enabled by spin anisotropy change across the SRT. The critical role of domain walls in generating topological textures and transformation without the requirement of external magnetic fields is revealed through experiments and micromagnetic simulation and agrees with the recently proposed theory about domain wall skyrmion^30–33^. Our results, therefore, point to a promising direction of controlling the spin topology by simply varying temperatures or electric current at zero field. The guided motion of skyrmions inside the domain wall and the contribution of partially compensated magnetization in the ferrimagnetic film will open great opportunities for a deep understanding of topological Hall effects and their application in electronics, spintronics storage. Given that domain walls are ubiquitous magnetic structures with potential fast DW dynamics, our findings provide critical insights in discovering, yet hitherto unknown, topological spin textures and magnetic phase transition in the domain wall.

## Methods

### Sample preparation and characterization

The ferrimagnetic thin films Pt (3 nm)/Gd_15+*x*_(Fe_94_Co_6_)_85−*x*_(*x* = 0.7,0.4,0.2,0)(40 nm)/Pt(3 nm) with different composition ratios of Gd to FeCo were grown by magnetron sputtering on 10-nm-thick Si_3_N_4_ membrane windows for direct TEM observation and simultaneously on Si/SiO_*x*_ wafers for physical property measurements. The higher composition of transition metal (FeCo) presents stronger PMA, therefore, four representative films with different SRT temperatures are selected. The magnetic hysteresis (M-H) loops of the Si/SiO_*x*_-based samples were measured at different temperatures using a superconducting quantum interference device magnetometer (SQUID). The amorphous state of GdFeCo thin film is characterized by electron diffraction.

### L-TEM observation

The magnetic domain structures were studied by using a JEOL-dedicated Lorentz TEM (JEOL2100F) equipped with a liquid-nitrogen or high-temperature TEM holder for temperature manipulation. No magnetic field is applied during the L-TEM observation due to the special design of the objective lens. There is about a 10 K temperature difference between L-TEM observation and the magnetic properties measurement in Gd_15+*x*_(Fe_94_Co_6_)_85−*x*_ (*x* = 0.7, 0.4) because the sample cannot be cooled first before heating up by using a high-temperature TEM holder. The magnetic domain wall contrast is imaged under the convergent or divergent electron beam due to the interaction of the electron beam with the in-plane magnetization. The under- and over-focal images were recorded by a charge-coupled device (CCD) camera to work out phase images and then the quantitative in-plane magnetization component on the basis of the TIE equation using commercial software QPt. The colors and arrows depict the magnitude and orientation of the in-plane magnetization according to the color wheel. The skyrmion manipulation behavior by an electric current is conducted using a double-tilt electrical TEM holder with two electrical conducting blocks at two sides of the TEM sample. The dc current was supplied using a source-measure unit instrument (Keithley 2601B).

### TIE analysis

The TIE equation is composed of the following two equations^48^:

2 $$\frac{2\pi }{\lambda }\frac{\partial I(x,y,z)}{\partial z}=-{\nabla }_{{{{{{\bf{xy}}}}}}}(I(x,y,z){\nabla }_{{{{{{\bf{xy}}}}}}}\varPhi (x,y,z))$$

3 $${\nabla }_{{{{{{\bf{xy}}}}}}}\varPhi (x,y,z)=-\frac{e}{\hslash }({{{{{\bf{M}}}}}}\times {{{{{\bf{n}}}}}})t$$

The first equation reveals the relationship between intensity *I*(*x,y,z*) and phase *Ф*(*x,y,z*). *λ* is the spectrally weighted mean wavelength of illumination. In our experiment, we put the under-focused and over-focused pictures in software QPt to find the value of $\frac{\partial I(x,y,z)}{\partial {{{{{\rm{z}}}}}}}$ to obtain the phase information. Then by using the second equation, the in-plane magnetization components (${{{{{\bf{M}}}}}}\times {{{{{\bf{n}}}}}}$) can be accordingly found. **n** is the unit vector along the beam direction, **M** is the magnetization vector and *t* is the local sample thickness.

### Micromagnetic simulation

Micromagnetic simulations are performed using the OOMMF^49^ to investigate the dependence of magnetic structures on parameters, such as PMA (*K*_u_) and saturated magnetization (*M*_s_) for a fixed exchange stiffness (*A* = 7 × 10^−12^ J/m). To aim at the magnetization evolution towards energy minimum for the given conditions, the ferrimagnetic sample is simplified as a ferromagnetic system with reduced saturation magnetization and uniform exchange term. The size of the thin plate GdFeCo film for simulation is about 1400 × 500 × 40 nm^3^ (the experimental thickness) with periodic boundary conditions in the *y-direction* and open boundary conditions in the *x-direction*. According to the simulation, the distance between each Bloch line at steady-state scales around 250 nm based on the experiment parameters (Supplementary Fig. 7), the *y* range used in our simulation is set as 500 nm which is enough for the stabilization of one meron pair. The mesh size is 2 × 2 × 5 nm^3^, which is much smaller than the typical exchange length and the skyrmion size, to ensure a balance between numerical accuracy and computational efficiency. In-plane spin anisotropy value of 2 × 10^3^ J/m^3^ along (100) direction is added to stabilize the domain wall during simulation. We then change the out-of-plane anisotropy and study the evolution of the magnetization configuration. We used an exchange stiffness parameter of about 7 pJ/m to make the domain width wider and simultaneously varied *M*_s_, *K*_u_ to study the domain evolution. The corresponding L-TEM image simulation is carried by the software called micromagnetic analysis to Lorentz TEM simulation (MALTS)^50^.

## Supplementary information

Supplementary information

Supplementary Video 1

Supplementary Video 2

Description of Additional Supplementary Files

## Data Availability

All the data that support the findings of this study are present in the paper and are available from the corresponding author upon reasonable request.
