# Supplementary material for: Field-free topological behavior in the magnetic domain wall of ferrimagnetic GdFeCo
Source: Nat Commun. 2021 Sep 23;12:5604. doi: 10.1038/s41467-021-25926-4 (PMC8460835; doi:10.1038/s41467-021-25926-4)
Supplement: Supplementary file 1 — Supplementary information [file 41467_2021_25926_MOESM1_ESM.pdf]

Supplementary Information for

Field-free topological behavior in the magnetic domain wall of ferrimagnetic GdFeCo

Zhuolin Li[1,2,#], Jian Su[1,2,#], Shi-Zeng Lin[3], Dan Liu[4], Yang Gao[5], Shouguo Wang[5], Hongxiang Wei[1], Tongyun Zhao[1], Ying Zhang[1,6,*], Jianwang Cai[1,2] and Baogen Shen[1,2]

[1] *Beijing National Laboratory for Condensed Matter Physics, Institute of Physics, Chinese Academy of Sciences, Beijing 100190, China*

[2] *School of Physical Sciences, University of Chinese Academy of Sciences, Beijing 100049, China*

[3] *Theoretical Division, Los Alamos National Laboratory, Los Alamos, New Mexico 87545, USA*

[4] *Department of Physics, Beijing Technology and Business University, Beijing 100048, China*

[5] *Institute of Advanced Materials, Beijing Normal University, Beijing 100875, China*

[6] *Songshan Lake Materials Laboratory, Dongguan, Guangdong 523808, China*

[#] These authors contributed to this work equally.

Corresponding authors: zhangy@iphy.ac.cn

Supplementary Note 1. Magnetic properties and magnetic domain images for GdFeCo ferrimagnetic films with varying RE-to-TM ratios in composition

The ferrimagnetic Pt(3nm)/$Gd_{15+x}(Fe_{94}Co_6)_{85-x}$ (x= 0.7, 0.4, 0.2, 0) (40nm)/Pt(3nm) thin film with different composition ratio of Gd to FeCo are grown by magnetron sputtering on 10 nm-thick Si_3N_4 membrane windows for direct L-TEM observation and simultaneously on Si/SiO_x wafers for magnetic property measurements. The normalized magnetic hysteresis (M-H) loops are measured at different temperatures using superconducting quantum interference device magnetometer (SQUID) as shown in Supplementary Fig. 1, indicating stronger perpendicular magnetic anisotropy (PMA) for higher composition ratio of transition metal (FeCo). Therefore, it will be crucial to establish a general mechanism to connect the desired domain-wall skyrmions or merons with the SRT by changing the ratio of Gd to FeCo during the fabrication of ferrimagnetic GdFeCo films.

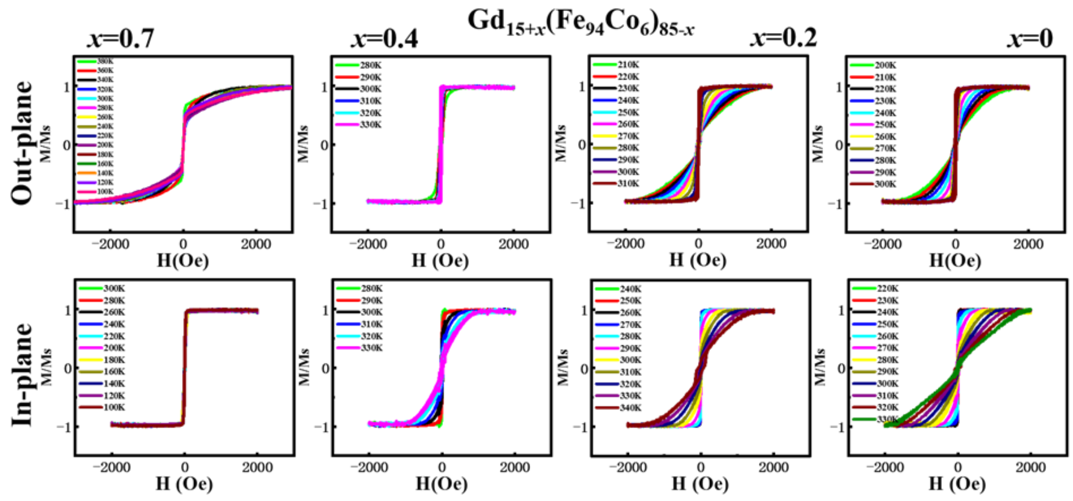

Supplementary Figure 1 | The in-plane and out-of-plane normalized M-H curves at different temperatures for $Gd_{15+x}(Fe_{94}Co_6)_{85-x}$ (x= 0.7, 0.4, 0.2, 0) ferrimagnetic films. Stronger PMA for higher composition ratio of transition metal (FeCo).

Supplementary Note 2. The magnetic domain evolution for GdFeCo ferrimagnetic films while in-situ changing the temperature at zero field.

The magnetic domain transformation is reversible with temperature change. The in-situ domain wall transformation has been manifested in $Gd_{15+x}(Fe_{94}Co_6)_{85-x}$ (x= 0.2) in

Fig. 1 of the main text. The similar transformation is also observed in series of samples but with different SRT temperatures. The topological evolution in the domain wall is studied in $Gd_{15+x}(Fe_{94}Co_{6})_{85-x}$ (x= 0) within a broader temperature range as shown in Supplementary Fig. 2. While increasing the temperature above SRT (273 K), the magnetization vector gradually rotates toward perpendicular direction and finally the system reaches stripe domain state at room temperature. In this process, the topological transformation from merons to skyrmions is observed in the domain wall. The SRT temperature can be adjusted to near room temperature (Supplementary Fig. 3 a-c) in $Gd_{15+x}(Fe_{94}Co_{6})_{85-x}$ (x= 0.4) to conveniently manipulate the transformation between merons and skyrmions in the domain wall. The domain wall merons are obtained at room temperature with surrounding in-plane ripple domains in $Gd_{15+x}(Fe_{94}Co_{6})_{85-x}$ (x= 0.7) when SRT temperature is near 358 K (Supplementary Fig. 3 d-f). It should be noted that the domain wall merons and the transformation into the skyrmions are equivalent for the domain wall with opposite contrast when increasing temperature (Supplementary Fig. 3). The coexistence of white and dark contrast domain wall is common in traditional magnets, indicating the neighbor domains with opposite in-plane magnetization. They have the equivalent opportunity to host topological objects and the characterization is the same except the contrast color is opposite.

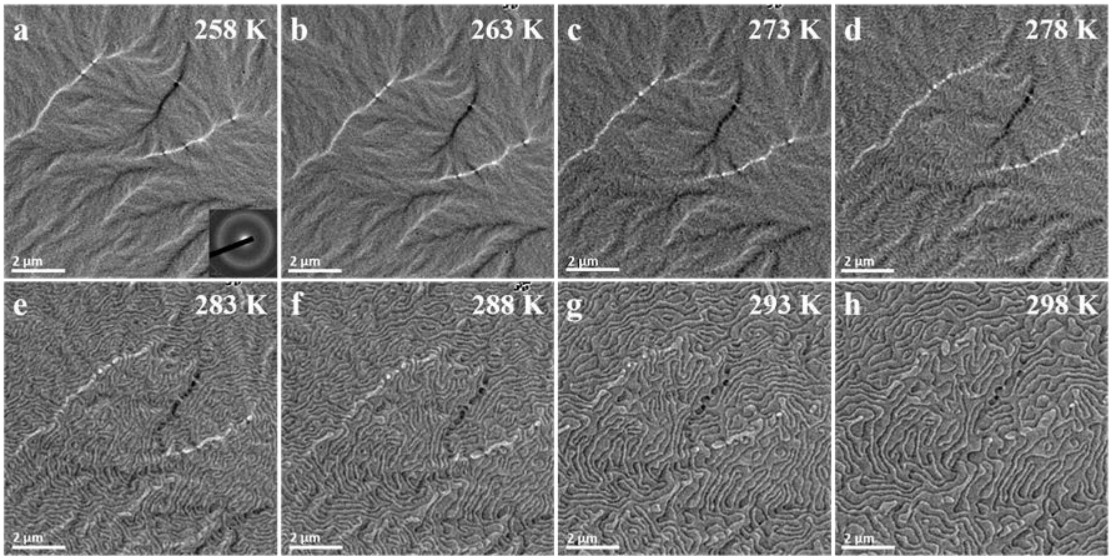

Supplementary Figure 2 | L-TEM images for magnetic domain evolution of $Gd_{15+x}(Fe_{94}Co_{6})_{85-x}$ (x= 0) while in-situ changing the temperature. The typical ripple domains with fluctuation of local in-plane magnetization change into out-of-plane stripe domains while increasing the temperaure. At the

same time, the domain wall undergoes the topological transformation from merons to skyrmions and then disappear. The amorphous diffraction partten is in the inset of **a**. The scare bar is 2 um.

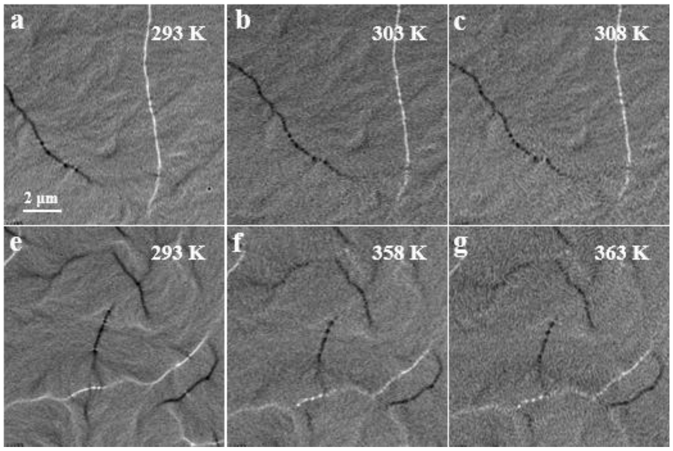

Supplementary Figure 3 | L-TEM images for magnetic domain evolution of GdFeCo while in-situ changing the temperature. a-c, Under-focused L-TEM images showing the transformation between merons and skyrmions near room temperature in $Gd_{15+x}(Fe_{94}Co_{6})_{85-x}$ (x= 0.4). The SRT at about 300 K. **d-f,** Under-focused L-TEM images showing meron pairs at room temperature by tuning the SRT transition temperature to about 358 K in $Gd_{15+x}(Fe_{94}Co_{6})_{85-x}$ (x= 0.7).

The temperature for the topology transformation in the domain wall tuned by the ratio of Gd to FeCo in GdFeCo films is summarized in Supplementary Fig. 4. The SRT temperature of $Gd_{15+x}(Fe_{94}Co_{6})_{85-x}$ decreases with smaller x as summarized in Supplementary Fig. 4a-c for x= 0.7, 0.4, 0, respectively. The magnetization at 300 K can be tuned from being in-plane dominant to out-of-plane dominant component by decreasing x as a consequence of increased perpendicular anisotropy. The SRT temperature is near 358 K for x= 0.7 according to L-TEM (Supplementary Fig. 2), which is beyond our magnetic property's measurement limit in Supplementary Fig. 4a. The corresponding magnetic domains in L-TEM images show in-plane ripple domains and meron pairs in the domain wall in Supplementary Fig. 4d and perpendicular stripe domains in Supplementary Fig. 4f. Therefore, the topological transformation between a pair of merons (N_s= ±1/2) with total topological number of ±1 and a single skyrmion with topological number of ±1, can be adjusted to near room temperature (Supplementary Fig. 4e) at zero magnetic field in $Gd_{15+x}(Fe_{94}Co_{6})_{85-x}$ (x= 0.4) for convenient applications.

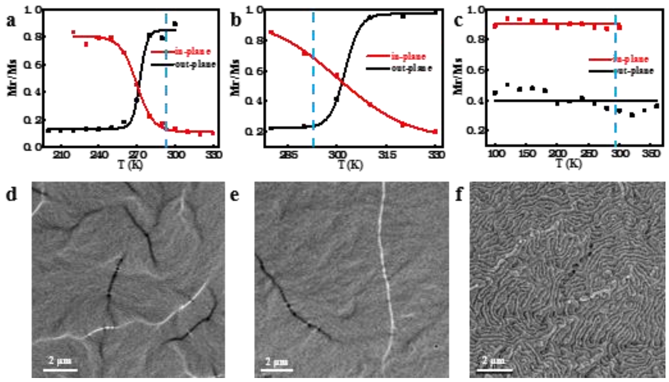

Supplementary Figure 4 | Tunable magnetic anisotropy and magnetic domains via changing the ratio of Gd to FeCo in GdFeCo films. a-c, The temperature dependence of the reduced in-plane and out-of-plane remnant magnetization acquired from M-H measurements in $Gd_{15+x}(Fe_{94}Co_{6})_{85-x}$ (x= 0.7, 0.4, 0), respectively. The SRT moves to lower temperature for higher composition ratio of transition metal (FeCo). **d-f**, The corresponding L-TEM images at 293 K for x= 0.7, 0.4, 0, showing typical ripple domains with dominant in-plane anisotropy and perpendicular stripe domains with out-of-plane anisotropy.

Supplementary Note 3. The spin configuration of the domain wall.

The Bloch-type domain wall is identified by tilting the sample[1]. The L-TEM images in Supplementary Fig. 5 demonstrate the contrast changes of the domain wall in $Gd_{15+x}(Fe_{94}Co_{6})_{85-x}$ for x= 0.4 (Supplementary Fig. 5a,b) and x= 0.2 (Supplementary Fig. 5c,d) while tilting the sample. The half black and half white contrast after tilting indicates the Bloch type domain wall due to the perpendicular-aligning antiparallel magnetization after tiltling. The Bloch type domain wall is further verified by micromagnetic simulations and L-TEM simulation, where the in-plane Bloch type domain wall and Néel type domain wall are compared in Supplementary Fig. 6. For the two types of domain walls at zero tilt angle, similar black contrast is observed. The contrast of the Bloch type domain wall changes into half black and half white (Supplementary Fig. 6c) while the Néel type stays the same (Supplementary Fig. 6f) after tilting the sample along y axis. Therefore, both the experiment and simulation results confirm the Bloch type domain wall.

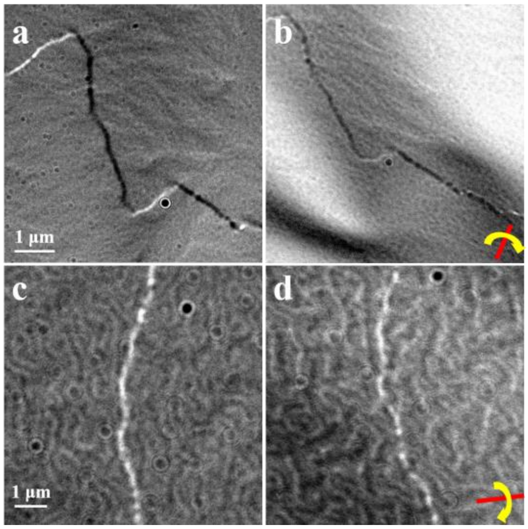

Supplementary Figure 5 | L-TEM images of domain walls to identify the domain wall type by tilting the sample. a, The domain wall contrast at zero angle in $Gd_{15+x}(Fe_{94}Co_6)_{85-x}$ for x= 0.4. **b,** At the tilted angle of -50 degrees. **c,** The domain wall contrast at zero angle in $Gd_{15+x}(Fe_{94}Co_6)_{85-x}$ for x= 0.2. **d,** At tilted angle of -40 degrees ('-' means anti-clockwise). The tilting axis is shown above.

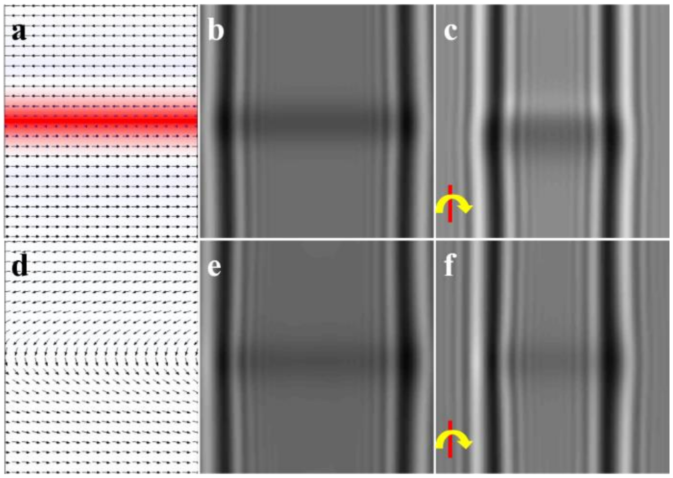

Supplementary Figure 6 | L-TEM simulated images of in-plane Bloch type and Néel typedomain walls. a, The micromagnetic simulation of Bloch type domain wall. L-TEM simulation images of Bloch type domain wall for different tilted angles, **b,** 0 degree, and **c,** -50 degrees. **d,** The micromagnetic simulation of Néel type domain wall after stablization. L-TEM simulation images of Néel type domain wall for different tilted angle, **e,** 0 degree, and **f,** -50 degrees. Tilting axis is along the y direction. The narrower width in **c** and **f** is caused by the rotation.

Supplementary Note 4. Estimate of DMI in GdFeCo.

DMI is generally present in magnets without inversion symmetry and is a key magnetic interaction for the generation of skyrmions. Normally, the amorphous GdFeCo is centrosymmetric, and therefore no DMI is allowed by symmetry. The reported DMI in

other GdFeCo compounds could be introduced by the interfacial SOT coupling[2] or the composition gradient[3] that breaks the inversion symmetry. The interfacial DMI should be weak in our system because of the symmetrical structure (GdFeCo sandwiched between two Pt layers) and the relatively thick thickness (40 nm). To make this point clear, Brillouin light scattering is conducted on our samples to measure the DMI. The schematic of the Brillouin light scattering measurement and the definition of incident angle θ is shown in Supplementary Fig. 7a. The frequency f dependence of the reflected light intensity at different incident angles demonstrate the two intensity peaks, representing the magnon creation and annihilation caused by incident light, respectively. Different incident angle stands for different spin wave length k. The frequency difference of the two intensity peaks for each k is extracted from Supplementary Fig. 7b and displayed in Supplementary Fig. 7c. The linear fitting line with zero slope demonstrates almost no DMI as shown in the representative $Gd_{15+x}(Fe_{94}Co_{6})_{85-x}$ (x= 0.2) since the value of slope is proportional to the value of DMI according to the formula

$$\Delta f(k) = \frac{2\gamma}{\pi M_s} Dk \tag{1}$$

where D is the DMI constant, γ is the gyromagnetic ratrio, Ms is the saturation magnetization[4]

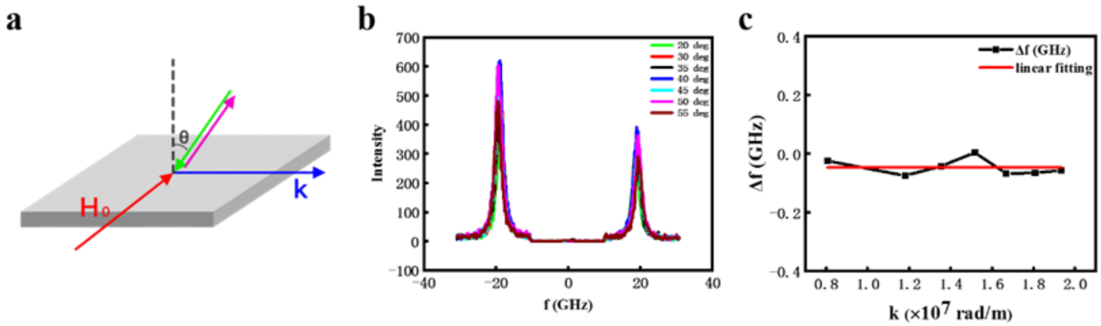

Supplementary Figure 7 | The Brillouin light scattering measurements of the representative sample $Gd_{15+x}(Fe_{94}Co_{6})_{85-x}$ (x= 0.2). a, The schematic of the Brillouin light scattering measurement and the definition of incident angle θ. The magnon wave length is denoted by k and the in-plane applied field is sketched by H_0. **b,** The frequency dependence of the reflected light intensity at different incident angles. **c,** The frequency difference of the two intensity peaks for each k and the comparison to the linear fitting. The linear fitting line with zero-slope demonstrating almost no DMI. The relationship between k and incident angle θ is $k = 4\pi \ sin\,\theta\,/\lambda$ with incident laser wavelength $\lambda = 532$ nm.

Moreover, the micromagnetic simulation of the spin configuration in Bloch lines demonstrate the appearance of Bloch lines without DMI (Supplementary Fig. 8a) and the specific chirality with DMI[5-7] (Supplementary Fig. 8b). The appearance of the Bloch line

corresponds well with our experimental results and is caused by the equal energy for the two chirality in centrosymmetric system. The swirling spin configuration for skyrmions and the meron pairs originate from the naturally helical spin orientation across the domain wall in correlation with the generation mechanism of domain wall topology by Bloch lines (Supplementary Fig. 9 and 10) in this centrosymmetric system, which is different from that in chiral magnets.

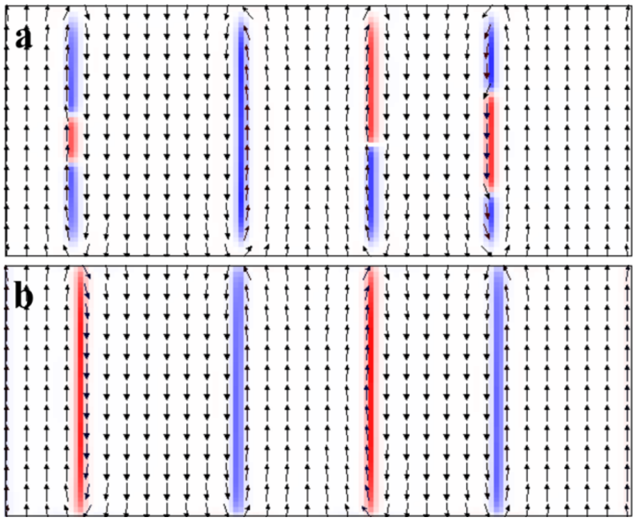

Supplementary Figure 8 | The micromagnetic simulation results of the same system with different value of DMI. a, 0 mJ/m^2 **b,** 0.5 mJ/m^2. The color red stands for the components in the positive z direction and blue stands for the components in the negative z direction.

Supplementary Note 5. The generation mechanism for the domain wall topology.

To understand the domain wall evolution, the magnetic parameters such as saturation magnetization M_s, and uniaxial perpendicular anisotropy K_u in $Gd_{15+x}(Fe_{94}Co_6)_{85-x}$ (x= 0.2) are extracted from above experiments for micromagnetic simulations in the Object Oriented Micromagnetic Framework (OOMMF) software. The overall domain structures is summarized in Fig. 3 of the main text by using a combination of experimental data and simulations. The meron pairs are generated from the fine structures with the right and left-hand Bloch wall pieces divided by high-density Bloch lines (BLs) (Supplementary Fig. 9-10).The distance between each Bloch line scales around 250 nm, which doesn't change much when y ranges from 500 nm to 2000 nm,

as shown in Supplementary Fig. 9. The distance corresponds well with the size of meron or width of domain wall. Supplementary Figure 10 clearly demonstrates the strong connections between meron pairs and BLs.

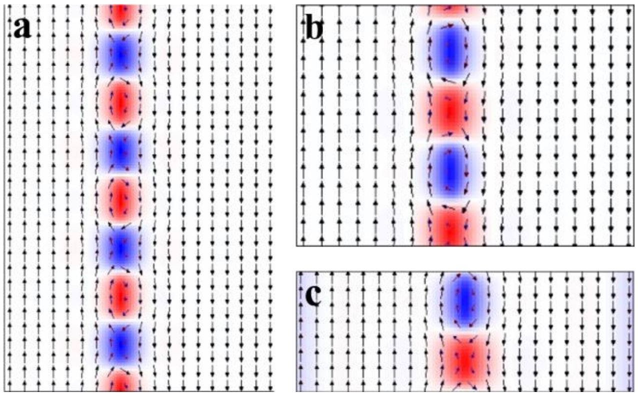

Supplementary Figure 9 | Micromagnetic simulation with different range of y. a, 2000 nm, **b,** 1000nm, **c,** 500nm. The parameters used in simulation are extracted from experiment at 270 K in $Gd_{15+x}(Fe_{94}Co_{6})_{85-x}$ (x= 0.2). The initial state is random magnetization.

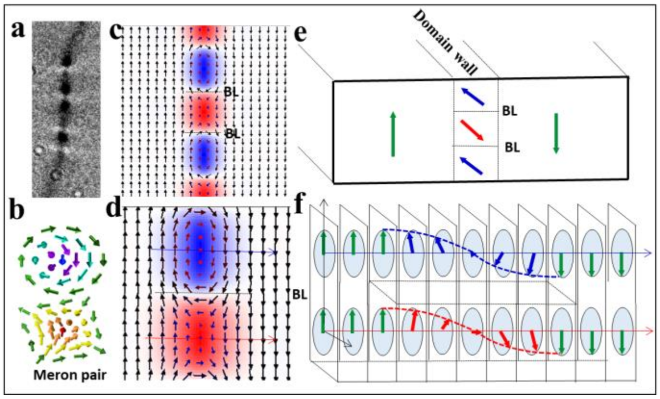

Supplementary Figure 10 | The generation mechanism for meron pairs. a, The L-TEM image with black and white contrast along the domain wall. **b,** The schematic spin configuration of meron pair. **c,** The simulated domain wall with periodic BLs separated. **d,** The simulated meron pair with one BL in the middle. **e,** The schematic 3-dimentional domain wall between antiparallel in-plane magnetization, demonstrating the strong spin correlations between meron pairs and BLs. **f,** The schematic 3-dimentional spin configuration, focusing on the spin configuration transition across the domain wall. The out-of-plane magnetization is represented by regions in red ($+m_z$) and blue ($-m_z$), whereas the in-plane magnetization is represented by white regions with black arrows.

We introduce the following spin anisotropies $H_A = -A_y S_y^2 - A_z S_z^2$ in the simulations. The spin anisotropy in H_A supports four degenerate domains which we denote by (±, ±). The first/second sign represents the *y/z* component of the spin in the domain. Domain walls can be formed between any pair of these four degenerate states. The simulated L-TEM

images for the spin configuration with parallel out-of-plane magnetization component near each side of the domain (Fig. 2 and 3 in the main text) agrees well with the experimental observation in Fig. 1 of the main text, where the white domain contrast disappears at skyrmion state. For an alternative possibility, an initial state with opposite z-components on the sides of the domain wall is shown in Supplementary Fig. 11b. The final state in the L-TEM simulation shows the spin configuration locating at the continuous domain wall with white and black contrast, which is different from experiments.

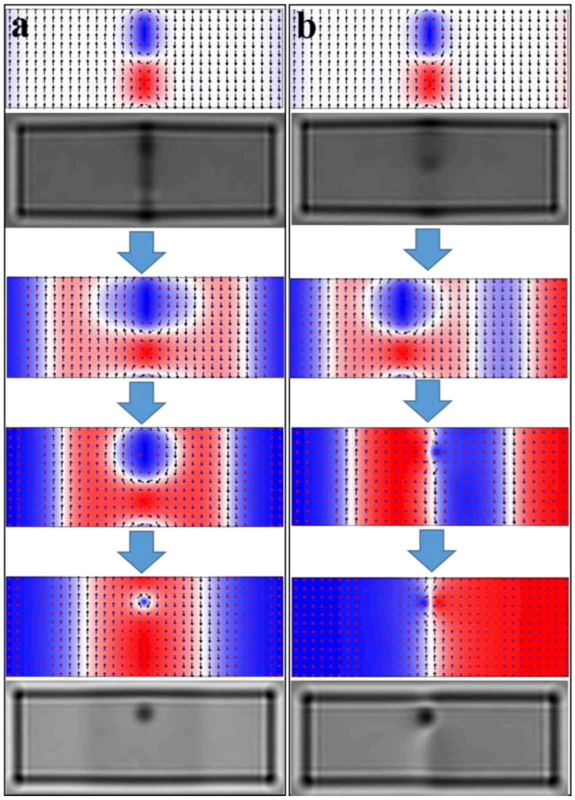

Supplementary Figure 11 | The micromagnetic analysis and L-TEM simulation for the domain wall with different spin configurations. a, The domain wall evolution and corresponding L-TEM contrast with the same z-component magnetization. The domain wall contrast evolution in agrees well with our LTEM images, with the white contrast diminish at skyrmion state. **b,** The opposite z-component magnetization on the sides of the domain wall. The simulation mimics the experimental temperature increase with varying the value of M_s and K_u. The additional white contrast domain wall results from the opposite z-component magnetization on the sides of the domain wall, which is not observed in our experiments.

Supplementary Note 6. The electric current manipulation on domain wall topology.

In these ferrimagnetic GdFeCo materials with partially compensated magnetic moments and distinctive domain wall topology, the dynamics of skyrmions under electric current is quite different from that of skyrmions in thin films. Here we simulate the dynamics of the domain wall skyrmion under the spin transfer torque (STT) effect in the area 1 μm x 1 μm x 40 nm with mesh size of 5 nm as shown in Supplementary video 1. The model we use is the LLG equation with adiabatic and non-adiabatic spin torques terms[8,9]:

$$\dot{\mathbf{m}} = \gamma_0 \mathbf{H} \times \mathbf{m} + \alpha \mathbf{m} \times \dot{\mathbf{m}} - (\mathbf{u} \cdot \nabla)\mathbf{m} + \beta \mathbf{m} \times [(\mathbf{u} \cdot \nabla)\mathbf{m}] \quad (2)$$

The meanings of the symbols are: γ_0 gyomagnetic constant, **H** micomagnetic effective field, **m** unit vector representing the local magnetization, α Gilbert damping constant, β non-adiabatic parameter, $\mathbf{u}$ the velocity of electrons. The amplitude of $\mathbf{u}$ is proportional to current density J and polarization P:

$$u = JPg\mu_B/(2eMs) \quad (3)$$

In our calculations, we choose M_s= 1 x 10^5 A/m, exchange integral A= 7 pJ/m, current polarization P= 0.4, damping coefficient α= 0.1 and nonadiabatic coefficient β= 0.04. The polarization P, damping coefficient α and nonadiabatic spin transfer torque coefficient β are default values provided by the solvers in OOMMF, which is applicable for most cases. To stabilize the domain wall skyrmion, an in-plane anisotropy energy with constant value of 1 x 10^4 J/m^3 along (100) direction and magnetic field with value of 100 Oe along (001) are applied. After being stabilized, the STT is added with the electron velocity of 100 m/s along (100) direction (left to right). The corresponding current density is of the order of 10^{11} A/m^2. The total duration of the current pulse is 5 ns. In the video, the skyrmion moves along the magnetic domain wall and the deflection angle is only 0.03 degree, demonstrating the potential elimination of skyrmion Hall effect due to the domain wall confinement. As for the experimental realization of skyrmion driven behavior, more difficult and critical situations should be considered. In previous experimentally studies, the micro-size skyrmions is easier to be driven with a patterned narrow path[10]. Therefore, nano-fabrication and pinning effect need to be paid attention in our upcoming work.

Here, prelimnary experiments on the transformation from merons to skyrmions and the skyrmion motion have been conducted by applying electric current in a double-tilt electrical TEM holder with two electrical conducting blocks at two sides of the TEM

sample as shown in the schematic illustration (Fig. 4 in the main text). The DC current was supplied by a source–measure unit instrument (Keithley 2601B). The preliminary experiments in uniform large samples have promisingly shown the skyrmion motion in the domain wall as presented in Supplementary video 1, 2. However, careful nano-fabrication and pinning effects should be considered for future study.

Supplementary References

1. Benitez, M., Hrabec, A., Mihai, A. et al. Magnetic microscopy and topological stability of homochiral Néel domain walls in a Pt/Co/AlOx trilayer. *Nat Commun* **6**, 8957 (2015)

2. Woo, S. et al. Current-driven dynamics and inhibition of the skyrmion Hall effect of ferrimagnetic skyrmions in GdFeCo films. *Nat. Commun.* **9**, 959 (2018).

3. Kim, D. et al. Bulk Dzyaloshinskii – Moriya interaction in amorphous ferrimagnetic alloys. *Nat. Mater.* **18**, 685–690 (2019).

4. Di, K. et al. Direct observation of the Dzyaloshinskii–Moriya interaction in a Pt/Co/Ni film. *Phys. Rev. Lett.* **114**, 047201 (2015).

5. Thiaville, A. The demagnetizing field inside a domain wall. *J. Magn. Magn. Mater.* **140**, 1877 (1995).

6. Malozemoff, A. P. and Slonczewski, J. C. Effect of Bloch lines on Magnetic Domain-Wall Mobility. *Phys. Rev. Lett.* **29**, 952 (1972).

7. Slonczewski, J. C. Theory of Bloch-line and Bloch-wall motion. *J. Appl. Phys.* **45**, 2705 (1974).

8. Thiaville, A., Nakatani, Y., Miltat, J. & Suzuki, Y. Micromagnetic understanding of current-driven domain wall motion in patterned nanowires. *Europhys.Lett.* **69**, 990–996 (2005).

9. Sampaio, J., Cros, V., Rohart, S., Thiaville, A. & Fert, A. Nucleation, stability and current-induced motion of isolated magnetic skyrmions in nanostructures. *Nature Nanotech.* **8**, 839–844 (2013).

10. Jiang, W. et al. Direct observation of the skyrmion Hall effect. *Nat. Phys.* **13**, 162–169 (2017).
