# Supplementary material for: Field-free topological behavior in the magnetic domain wall of ferrimagnetic GdFeCo
Source: Nat Commun. 2021 Sep 23;12:5604. doi: 10.1038/s41467-021-25926-4 (PMC8460835; doi:10.1038/s41467-021-25926-4)
Supplement: Supplementary file 4 — Description of Additional Supplementary Files [file 41467_2021_25926_MOESM4_ESM.pdf]

Title: Supplementary Movie 1:
Description: The simulated domain wall topology dynamics driven by the electric current.

Title: Supplementary Movie 2:
Description: The experimental domain wall topology dynamics driven by the electric current at room temperature.
